# Public knowledge and beliefs about the irritable bowel syndrome - results from the SOMA.SOC study

**DOI:** 10.1186/s12889-024-17784-8

**Published:** 2024-01-18

**Authors:** Olaf von dem Knesebeck, Bernd Löwe, Daniel Lüdecke, Johanna S. Bobardt, Rieke Barbek

**Affiliations:** 1 Institute of Medical Sociology, University Medical Center Hamburg-Eppendorf (UKE), Martinstr.52, 20246 Hamburg, Germany; 2 Department of Psychosomatic Medicine and Psychotherapy, UKE, Hamburg, Germany; 3 Department of General Practice and Primary Care, UKE, Hamburg, Germany

**Keywords:** Irritable bowel syndrome, Knowledge, Beliefs, Health literacy, SOMA.SOC

## Abstract

**Background:**

Despite the epidemiological and economic relevance of the irritable bowel syndrome (IBS), there is a lack of research on what the general public knows and thinks about this condition (IBS literacy). Therefore, the aim of this study was to explore public knowledge and beliefs about IBS in Germany. Moreover, associations of knowledge and beliefs about IBS with socio-demographic characteristics as well as illness and treatment experiences were analysed.

**Methods:**

Analyses made use of a national telephone survey (*N* = 1,205). A carefully developed vignette describing a person with typical symptoms of IBS was presented. Respondents were then asked to name the disease in question and beliefs about causes and treatment options were assessed. For the analyses respondents were divided into three groups: (1) people who never had IBS symptoms, (2) people who had or have IBS symptoms but never were in treatment and (3) individuals who reported to be or have been treated for IBS symptoms.

**Results:**

Less than 4% of the respondents recognized IBS after presentation of the vignette. About 75% positively evaluated treatability while psychotherapy was evaluated more effective than medication. Stress and unhealthy lifestyle were the most frequently endorsed possible causes of the presented IBS symptoms. There were variations in knowledge and beliefs about IBS according to age, gender, and education. We found minor differences in beliefs and knowledge between individuals who had or have symptoms but never were in treatment and those without respective illness experience. Respondents with illness/treatment experiences rated their knowledge significantly better than those without any experiences.

**Conclusions:**

Results indicate low levels of public knowledge about IBS regarding illness recognition in Germany. A majority disagreed that they have good knowledge about IBS symptoms. Against this background, it seems reasonable to develop and test interventions to improve IBS literacy by increasing knowledge about symptoms, causes and treatment options.

**Supplementary Information:**

The online version contains supplementary material available at 10.1186/s12889-024-17784-8.

## Background

The concept of health literacy refers to people’s knowledge, motivation and competencies of accessing, understanding, appraising and applying health-related information in order to make judgments and decisions in everyday life concerning health care, disease prevention, and health promotion [1]. Studies from different countries indicated that between one and two thirds of the population report inadequate or problematic health literacy [2]. Such low levels of health literacy can negatively affect health, health or illness related behaviour, and the adequate utilization of health care [3]. Low levels were more often observed among individuals with advanced age, low education, and a history of migration [3, 4], suggesting social inequalities in health literacy.

As the definition of health literacy is rather general, more specific conceptualizations have been developed, particularly related to knowledge and beliefs about specific diseases (e.g. cancer literacy [5] or depression literacy [6]). In respective empirical studies several components were assessed, including the ability to recognize the specific disorder as well as knowledge and beliefs about causes and treatment options [7]. Some of these studies also considered the role of illness and treatment experiences for disease specific literacies. In this regard, it is assumed that people who have a personal illness or treatment history show higher levels of literacies. Corresponding empirical research yielded inconsistent results and mostly related to mental illnesses [8–11].

Irritable bowel syndrome (IBS) is a common condition that affects the digestive system [12, 13]. Recent estimates of the worldwide prevalence in the general population vary between about 4% (according to the Rome IV criteria) and about 10% (according to the Rome III criteria), with women and people younger than 40 years being more often affected [13, 14]. IBS has a multi-factorial pathophysiology involving biological, social, psychological, and iatrogenic factors [12]. It causes various chronic symptoms (e.g. abdominal pain, diarrhoea, bloating) that can have a considerable negative impact on the quality of life of afflicted individuals. Many other functional gastrointestinal disorders have symptoms that overlap with IBS symptoms which makes the diagnosis a challenge [15–17]. The majority of patients is diagnosed in primary care [15, 17, 18]. There are several treatment options that can help to control the symptoms (e.g. medication, diet change, and psychotherapy). Despite the epidemiological and economic relevance of IBS [19, 20], there are only a few studies on public knowledge and beliefs about IBS (IBS literacy). A population survey from the United States demonstrated a lack of knowledge about IBS [21]. This was supported by a study from Saudi Arabia in which participants showed a poor understanding of the causes and complications of IBS [22]. Overall, there is not much known about the magnitude and predictors of IBS literacy.

Against this background, the aim of this study was to explore public knowledge and beliefs about IBS in Germany. More specifically, associations of knowledge and beliefs about IBS with socio-demographic characteristics as well as illness and treatment experiences were analysed. In terms of the latter, three groups were distinguished: people who never had IBS symptoms, people who had or have IBS symptoms but never were in treatment and individuals who reported to be or have been treated for IBS symptoms.

## Methods

### Study design and sample

Analyses made use of cross-sectional data collected between March and May 2022 via a telephone survey (computer assisted telephone interview) of the adult population (age ≥ 18 years) living in Germany. About 70% of the sample was randomly drawn from all registered private telephone numbers. To ensure inclusion of ex-directory households (landline numbers), computer-generated numbers were used additionally. The other 30% of the sample consisted of randomly generated mobile phone numbers. To randomly select participants in the households, the Kish-Selection Grid was applied [23]. With this method, all individuals in a household have an equal chance of selection. Oral informed consent was given at the beginning of the interview. In total, *N* = 2,413 individuals participated in the survey, reflecting a response rate of 45%. To obtain a representative sample of the adult population living in Germany, the data set was weighted. Therefore, distribution of socio-demographic characteristics of the sample (age, gender, and education) is similar to the general adult population in Germany. The survey is part of a project on social inequalities in aggravating factors of persistent somatic symptoms (SOMA.SOC) which is embedded in the Research Unit 5211 “Persistent SOMAtic Symptoms ACROSS Diseases: From Risk Factors to Modification (SOMACROSS)” [24]. Methodological details about the SOMA.SOC study were described in a study protocol [25]. The study design was approved by the Ethics Commission of the Hamburg Medical Chamber (No. 2020-10194-BO-ff).

### Vignettes

At the beginning of the interviews, a vignette describing a person with typical symptoms of IBS or fatigue was presented to the respondents in the survey. We chose these two specific conditions as they appear relatively frequently in the German population and other projects of the research unit also focussed on IBS and fatigue. Vignettes were carefully developed with the input of clinicians (colleagues of the SOMACROSS research unit, i.e. specialists from gastroenterology, psychosomatic medicine, general and internal medicine) considering typical symptoms described in the International Classification of Diseases (ICD 10 [26]). One of the two vignettes (IBS or fatigue) was randomly assigned to half of the sample respectively. As the following analyses will focus on IBS, half of the total sample (*n* = 1,205) will be used. In terms of the IBS vignette, different symptoms like abdominal cramps, flatulence and diarrhoea were described (please see Appendix). The vignettes were audio-recorded with a trained speaker with a clear voice. In order to increase reliability and to neutralize possible interviewer-associated effects, the file was presented to the respondents directly from the computer via telephone line. Vignettes were varied according to sex (male/female), occupational status (lawyer/cleaner), and migration status (yes/no). Thus, eight different IBS vignettes were used that each were randomly assigned to about 150 respondents (i.e. about 12.5% of the analysed sample). Sample size was targeted on detection of small to medium differences between the vignettes [25]. For the present analyses, the eight vignettes were pooled. We used unlabelled vignettes, i.e. the respondents were not informed that the person in the vignette had IBS.

### Measures

In terms of knowledge and beliefs about IBS symptoms, the following indicators were assessed after presentation of the vignette: First, the respondents were asked for their opinion on what disease the person in the vignette has. Answers to this open-ended question were discussed and coded by two researchers so that respondents were identified who recognized IBS. Based on previous studies [8, 27], causal attributions for the IBS symptoms were assessed afterwards. In this regard, respondents were asked to evaluate four potential causes (heredity, misinterpretation of body signals, unhealthy lifestyle, family or work stress) using a 4-point scale that ranged from “completely agree” to “completely disagree”. Furthermore, they were asked to assess, to what extent treatment for such symptoms is helpful in general on a scale ranging from 0 (“not at all”) to 10 (“very good”). This was followed by questions on how effective two specific options (medication and psychotherapy) are for treatment (“not at all”, “rather not”, “rather”, “very effective”). The self-assessed overall knowledge was measured by the item “I know a lot about symptoms like those of Mrs./Mr. E.”. Again, a 4-point scale was used ranging from “completely agree” to “completely disagree”.

Additionally, the respondents were asked whether they have ever been afflicted by symptoms like those presented in the vignette (“yes”/”no”) and if yes, whether they have been treated for these symptoms (“yes”/”no”). Accordingly, respondents were divided into three groups: (1) people who never had IBS symptoms, (2) people who had or have IBS symptoms but never were in treatment, and (3) individuals who reported to be or having been treated for IBS symptoms. Finally, age, gender, and education were assessed as socio-demographic characteristics.

### Analyses

Descriptive analyses were conducted to get a first overview on the three groups and the beliefs about IBS symptoms. To test for between-group differences, χ2-tests were performed. In order to explore associations between illness/treatment experiences and indicators of knowledge and beliefs, binary logistic regression analyses were carried out, adjusted for gender, age and education. To this end, for causal attributions and the item on overall knowledge, response categories were dichotomized, combining the respondents who (completely and rather) agreed in one group and those who (completely and rather) disagreed in another. Also the two items measuring effectiveness of the two treatment options were dichotomized by combining the first two (not at all effective/rather not effective) and the last two response options (rather/very effective). In terms of treatment in general, respondents who rated helpfulness with values from 0 to 4 will be compared to those with values from 5 to 10. Odds ratios, 95% confidence intervals and significances are displayed. The significance level for *p*-values was set at *p* < 0.05. Statistical procedures were performed with the statistical program package R 4.3 [28].

## Results

In the analysed sample, proportion of female and male respondents was similar (Table 1). Mean age of participants was 51.5 years. About one third of respondents had a low (≤ 9 years), medium (10 years) or high education (≥ 12 years), each. 64.6% of the respondents reported that they never had symptoms like those presented in the vignette, while 15.8% said that they are or have been afflicted by such symptoms but never received treatment, and 19.6% reported to be or having been treated for such symptoms. There were significant age differences regarding symptom and treatment experiences.

**Table 1:** Socio-demographic characteristics of the sample according to illness and treatment experiences

	Total (*N* = 1,205, 100%)	Never afflicted by IBS symptoms* (*n* = 778, 64.6%)	Afflicted by IBS symptoms*, no treatment** (*n* = 191, 15.8%)	Afflicted by IBS symptoms*, treatment** (*n* = 236, 19.6%)	*p*
Female (%)	49.5	48.1	56.8	48.2	0.100^1^
Age (mean, SD)	51.5 (18.5)	53.1 (18.6)	46.7 (19.0)	50.0 (17.1)	0.003^2^
Education (%)					0.231^1^
Low (≤ 9 years) Middle (10 years) High (≥ 12 years)	32.0 30.7 37.3	31.4 29.2 39.4	35.9 31.4 32.7	30.8 35.1 34.1	

IBS = irritable bowel syndrome, ^1^ Chi-square test, ^2^ Mann Whitney test

* “Have you ever been afflicted by symptoms like those presented in the vignette?” (yes/no)

** “If yes, have you been treated for these symptoms?” (yes/no)

About 4% of the participants correctly recognized IBS after presentation of the vignette (Table 2). This rate was significantly higher among females and respondents with high education. About three quarters positively evaluated treatability. Again, there were significant differences according to gender and education. About 64% evaluated medication rather or very effective to treat IBS symptoms. In terms of psychotherapy, this rate was about 75%. Regarding causal attributions, 38.5% rather or completely agreed that heredity is a possible cause. Agreement was more pronounced for a misinterpretation of body signals (46.1%). In this regard, there was a positive age gradient and a negative education gradient. Family or work stress (83.9%) and unhealthy lifestyle (65.9%) were the most frequently attributed possible causes of the presented IBS symptoms. Less than one third of the respondents (30.7%) agreed that they have a good knowledge about symptoms like those presented in the vignette. Agreement was significantly less pronounced among older and low educated participants.

**Table 2:** Knowledge and beliefs about irritable bowel syndrome (IBS) according to socio-demographic characteristics of the sample (*N* = 1,205)

	Total	Age groups				Gender			Education			
		18–40 *n* = 378	41–60 *n* = 428	61+ *n* = 398	*p* ^*^	Female *n* = 608	Male *n* = 595	*p* ^*^	Low *n* = 376	Middle *n* = 361	High *n* = 442	*p* ^*^
Recognition of IBS (%)	3.7	3.0	4.3	3.6	0.612	5.0	2.3	**0.013**	1.8	3.8	5.4	**0.029**
Treatment is helpful (0–10; values ≥ 5, %)	76.2	77.5	73.6	77.6	0.311	81. 0	71.5	**< 0.001**	71.6	79.2	80.0	**0.010**
Treatment (rather/very effective, %)												
Medication	63.8	64.4	59.0	68.3	**0.027**	61.6	66.0	0.132	65.1	63.6	63.3	0.858
Psychotherapy	74.6	72.3	75.0	76.5	0.418	78. 7	70.6	**0.002**	76.1	72.2	76.1	0.370
Possible causes (rather/completely agree, %)												
Heredity	38.5	38.5	37.8	39.2	0.914	37.2	39.7	0.406	38.6	38.2	38.3	0.996
Misinterpretation of body signals	46.1	36.5	46.5	55.3	**< 0.001**	47.3	45.0	0.469	58.2	43.3	38.5	**< 0.001**
Unhealthy lifestyle	65.9	72.4	60.8	65.0	**0.003**	59.0	72.6	**< 0.001**	61.9	67.6	67.6	0.167
Family or work stress	83.9	86.5	82.8	82.4	0.239	85.8	82.0	0.085	79.9	87.0	85.6	**0.024**
Good knowledge about symptoms (rather/completely agree, %)	30.7	39.1	30.3	23.1	**< 0.001**	33.2	28,3	0.072	24.8	35.4	32.3	**0.006**

^*^ Significance of Chi-square

Table 3 shows associations of knowledge and beliefs about IBS symptoms with illness and treatment experiences. Recognition of IBS was not significantly associated with illness and treatment experiences. Respondents who were in treatment for IBS symptoms were two times more likely to generally assess treatment as helpful (odds ratio 1.99) compared to those who never had IBS symptoms. However, no significant associations were observed for beliefs about the effectiveness of medication or psychotherapy. In terms of causal attributions, persons who had been in treatment were more likely to endorse heredity, misinterpretation of body signals, and stress as potential causes for IBS symptoms. There were strong positive associations between perceived knowledge about symptoms and illness as well as treatment experiences.

**Table 3:** Illness/treatment experiences and knowledge/beliefs about irritable bowel syndrome (IBS): odds ratios (95% CI)^a^, and significances (*N* = 1,205)

	Afflicted by IBS symptoms, no treatment^b^	Afflicted by IBS symptoms, treatment^b^
Recognition of IBS	1.64 (0.71–3.49)	1.06 (0.44–2.29)
Treatment is helpful (0–10; values > 5)	0.79 (0.54–1.16)	1.99 (1.32–3.01)**
Treatment (rather/very effective)		
Medication Psychotherapy	0.75 (0.53–1.07) 0.98 (0.67–1.45)	0.73 (0.54-1.00) 1.07 (0.76–1.51)
Possible causes (rather/completely agree)		
Heredity Misinterpretation of body signals Unhealthy lifestyle Family or work stress	1.15 (0.81–1.63) 1.03 (0.73–1.45) 0.91 (0.64–1.29) 1.04 (0.66–1.63)	1.40 (1.03–1.91)* 2.03 (1.49–2.78)*** 1.06 (0.77–1.47) 1.64 (1.03–2.62)*
Good knowledge about symptoms (rather/completely agree)	6.55 (4.57–9.40)***	8.96 (6.40-12.54)***

^a^ adjusted for gender, age and education

^b^ reference category: never afflicted by IBS symptoms

* *p* < 0.05, ** *p* < 0.01, *** *p* < 0.001

## Discussion

This is one of the first studies investigating public knowledge and beliefs about IBS. Based on a representative population sample in Germany, we found that less than 4% of the respondents recognized IBS after presentation of a vignette which was developed with the input of clinicians and included typical symptoms. About three quarters positively evaluated treatability while psychotherapy was evaluated more effective than medication. Stress and unhealthy lifestyle were the most frequently endorsed possible causes of the presented IBS symptoms. There were variations in knowledge and beliefs about IBS according to age, gender, and education. Socio-demographic differences (especially regarding age and education) were most pronounced in causal attributions for IBS. We additionally explored the role of illness and treatment experiences for IBS literacy. In this regard, we found minor differences in beliefs and knowledge between individuals who had or have symptoms but had never been treated and those without respective illness experience. Respondents with treatment experience were more likely to positively evaluate treatability and differed in causal attributions compared to those who had no experiences with IBS symptoms. There were no significant differences between the three groups regarding illness recognition and beliefs about the effectiveness of treatment options (medication, psychotherapy). Overall, respondents with illness/treatment experiences rated their knowledge significantly better than those without any experiences.

In our study, 15.8% of the respondents stated that they are or have been afflicted by symptoms like those presented in the vignette but never were in treatment, and 19.6% reported to be or having been treated for such symptoms. Due to the vignette design, these rates cannot be interpreted as prevalence estimations of IBS. Respondents were not informed about the diagnosis, and thus, their answers referred to the described symptoms in the vignette and not to IBS as a diagnosis. Symptom and treatment experiences did not differ according to education, while female respondents were slightly overrepresented among those afflicted, and respondents younger than 50 years more often reported symptom and treatment experiences. These results on socio-demographic variations are largely in line with epidemiological studies on IBS [19, 29].

Knowledge about IBS was less pronounced among older respondents, men, and people with low education. This was the case for the correct recognition of IBS as well as the self-evaluation of the overall knowledge about symptoms. Such socio-demographic disparities were also found in studies on general health literacy [3]. In terms of beliefs, there were marked differences in causal attributions especially according to age and education. Agreement that misinterpretation of body signals is a possible cause for the presented IBS symptoms was positively associated with age while there was a negative educational gradient. This indicator of causal attribution was selected to address somatosensory amplification as an important perceptual mechanism for persistent somatic symptoms [30] that was found to strengthen the belief about the effectiveness of psychotherapy in case of somatic symptom disorder [27].

In our study, recognition of IBS was not significantly associated with illness and treatment experiences. In other words, similar to those who never were afflicted by IBS symptoms, only a small number of respondents with illness and treatment experiences correctly recognized IBS after presentation of the vignette. This is remarkable as it was assumed that people who have a personal illness or treatment history show higher levels of literacies [7]. Some authors suspected that there are deficits in the diagnostic procedures and in the communication of the diagnosis among general practitioners [31] so that experienced patients may not be better informed. In this regard, a review showed that relatively few primary care physicians were aware of the formal diagnostic criteria for IBS but most could recognise the key IBS symptoms [32]. It has been highlighted that the diagnosis of IBS should be clearly communicated and explained to the patients [15, 17, 33]. “General practitioners’ key skills, especially in relation to chronic disorders such as IBS, are to make a positive diagnosis, including providing a simple explanation of the pathophysiology underlying the symptoms, clarifying the patient’s main concerns and managing current symptoms in the wider context of the patient’s life.” [17: 1221].

On the other hand, respondents with illness/treatment experiences were six to nine times more likely to agree that they have good knowledge about IBS symptoms than those without any experiences. Previous studies on illness recognition did not focus on IBS but, for example on mental health literacy and yielded mixed results. In a German study, respondents with experience of treatment for depression were more likely to correctly recognize the disorder compared to those who never were afflicted [8]. However, respondents who had a history of depression but not sought help did not show better rates of illness recognition. In a Swedish study, two thirds failed to recognize depression in a vignette [11]. Recognition did not differ between mentally healthy persons and persons with symptoms of mental illness with and without treatment contact. In terms of evaluation of treatability, illness experience alone did not make a difference, while a treatment history was positively associated. A similar pattern was observed for causal attributions: Associations with illness experiences were less pronounced than with treatment experiences. This pattern was also found in a study on mental health literacy [8].

There are some methodological aspects that must be considered when interpreting the present findings. Although a response rate of 45% seems acceptable, a selection bias due to non-response (refusal or non-availability) cannot be ruled out. We used vignettes which is considered a useful approach for studies on health literacy. Although these vignettes were carefully developed with the input of various clinicians, they had to be short and concise to be included in a telephone survey. Moreover, the IBS symptoms described in the vignettes can also reflect other functional gastrointestinal disorders due to an overlap of symptoms. This may be another explanation for the low proportion of respondents who recognized IBS. As outlined above, we used unlabelled vignettes, i.e. the respondents were not informed that the person in the vignette had IBS. Therefore, expressed knowledge and beliefs referred to the described symptoms and not to IBS as a diagnosis. Furthermore, the three groups regarding illness and treatment experiences also referred to the symptoms depicted in the vignette and were built based on self-reports. In terms of indicators for IBS literacy, interpretation is ambiguous in some cases because it is difficult to evaluate which beliefs about treatment options or causes of IBS are ‘correct’. Moreover, as we dichotomized the variables for the analyses, results on associations in a way are crude. We decided to proceed like this and use logistic regression models for the sake of clearness and due to the response scales of the IBS literacy indicators.

## Conclusions

Results indicate low levels of public knowledge about IBS regarding illness recognition in Germany. Recognition of IBS was not significantly associated with illness and treatment experiences. A majority of about 70% disagreed that they have good knowledge about symptoms. Against this background, it seems reasonable to develop and test interventions to improve knowledge about symptoms, causes and treatment options. Regarding general health literacy, national action plans for improvement have been developed in some countries [34, 35]. These action plans also contain recommendations that can be used to improve IBS literacy (e.g. establish user-friendly health information, enhance comprehensible communication between health professions and users, or strengthen the self-management ability of people with (chronic) diseases [35]). In terms of the latter measure, specific educational interventions for IBS patients have been developed and tested [36, 37]. Our results underline that it is important to consider social inequalities when such interventions are implemented.

### Electronic supplementary material

Below is the link to the electronic supplementary material.

Supplementary Material 1

## Data Availability

The dataset used is available from the corresponding author on reasonable request.

## Abbreviations

IBS: Irritable bowel syndrome

## References

[1] Sørensen K, Van den Broucke S, Fullam J, Doyle G, Pelikan J, Slonska Z (2012). Health literacy and public health: a systematic review and integration of definitions and models. BMC Public Health.

[2] Sørensen K, Pelikan JM, Röthlin F, Ganahl K, Slonska Z, Doyle G (2015). Health literacy in Europe: comparative results of the European health literacy survey (HLS-EU). Eur J Public Health.

[3] Schaeffer D, Berens EM, Vogt D (2017). Health literacy in the German population. Dtsch Arztebl Int.

[4] Knesebeck Ovd, Koens S, Schäfer I, Strauss A, Klein J (2022). Public knowledge about emergency care– results of a population survey from Germany. Front Public Health.

[5] Sørensen K, Makaroff LE, Myers L, Robinson P, Henning GJ, Gunther CE (2020). The call for a strategic framework to improve cancer literacy in Europe. Arch Public Health.

[6] Wang JL, Adair C, Fick G, Lai D, Evans B, Perry BW (2007). Depression literacy in Alberta: findings from a general population sample. Can J Psychiatry.

[7] Jorm AF (2000). Mental health literacy. Public knowledge and beliefs about mental disorders. Brit J Psychiatry.

[8] Mnich E, Makowski AC, Lambert M, Angermeyer MC, Knesebeck O (2014). Beliefs about depression - do affliction and treatment experience matter? Results of a population survey from Germany. J Affect Disord.

[9] Tomczyk S, Muehlan H, Freitag S, Stolzenburg S, Schomerus G, Schmidt S (2018). Is knowledge half the battle? The role of depression literacy in help-seeking among a non-clinical sample of adults with currently untreated mental health problems. J Affect Disord.

[10] Lauber C, Ajdacic-Gross V, Fritschi N, Stulz N, Rössler W (2005). Mental health literacy in an educational elite– an online survey among university students. BMC Public Health.

[11] Dahlberg KM, Waern M, Rundeson B (2008). Mental health literacy and attitudes in a Swedish community sample– investigating the role of personal experience of mental health care. BMC Public Health.

[12] Löwe B, Lohse A, Andresen V, Vettorazzi E, Rose M, Broicher W (2016). The development of irritable bowel syndrome: a prospective community-based cohort study. Am J Gastroenterol.

[13] Sperber AD, Bangdiwala SI, Drossman DA, Ghoshal UC, Simren M, Tack J (2021). Worldwide prevalence and burden of functional gastrointestinal disorders, results of Rome Foundation Global Study. Gastroenterol.

[14] Oka P, Parr H, Barberio B, Black CJ, Savarino EV, Ford AC (2020). Global prevalence of irritable bowel syndrome according to Rome III or IV criteria: a systematic review and meta-analysis. Lancet Gastroenterol Hepatol.

[15] Bobardt J, Freitag M (2023). Das Reizdarmsyndrom in Der hausärztlichen Praxis: Update Der S3-Leitline Reizdarmsyndrom. Z Allg Med.

[16] Schmulson MJ, Drossman DA (2017). What is new in Rome IV. J Neurogastroenterol Motil.

[17] Vasant DH, Paine PA, Black CJ, Houghton LA, Everitt HA, Corsetti M (2021). British Society of Gastroenterology guidelines on the management of irritable bowel syndrome. Gut.

[18] Layer P, Andresen V, Allescher H, Bischoff SC, Claßen M, Elsenbruch S (2021). Update S3-Leitlinie Reizdarmsyndrom: definition, Pathophysiologie, Diagnostik Und Therapie. Gemeinsame Leitlinie Der Deutschen Gesellschaft für Gastroenterologie, Verdauungs- Und Stoffwechselkrankheiten (DGVS) Und Der Deutschen Gesellschaft für Neurogastroenterologie Und Motilität (DGNM). Z Gastroenterol.

[19] Lovell RM, Ford AC (2012). Global prevalence of and risk factors for irritable bowel syndrome: a meta-analysis. Clin Gastroenterol Hepatol.

[20] Bosman MH, Weerts ZZ, Snijkers JTW, Vork L, Mujagic Z, Masclee AAM (2023). The socioeconomic impact of irritable bowel syndrome: an analysis of direct and indirect health care costs. Clin Gastroenterol Hepatol.

[21] Verne GN (2004). The public awareness of the prevalence and impact of irritable bowel syndrome in the United States. J Clin Gastroenterol.

[22] Qari YA (2016). Perception and reality—a study of public knowledge and perceptions about irritable bowel syndrome. Saudi J Intern Med.

[23] Kish L (1949). A procedure for objective Respondent selection within the household. J Am Stat Assoc.

[24] Löwe B, Andresen V, Van den Bergh O, Huber T, Knesebeck Ovd, Lohse AW (2022). Persistent SOMAtic symptoms ACROSS diseases - from risk factors to modification: Scientific Framework and Overarching Protocol of the Interdisciplinary SOMACROSS Research Unit (RU 5211). BMJ Open.

[25] Knesebeck Ovd, Barbek R, Makowski A (2023). Social inequalities in aggravating factors of somatic symptom persistence (SOMA.SOC): study protocol of a mixed method observational study focussing on irritable bowel syndrome and fatigue. BMJ Open.

[26] DIMDI. (2019). ICD-10-WHO Version. 2019. https://www.dimdi.de/static/de/klassifikationen/icd/icd-10-who/kode-suche/htmlamtl2019/ [Access July 6 2023].

[27] Knesebeck Ovd, Lehmann M, Löwe B, Lüdecke D (2020). Causal attributions for somatic symptom disorders– results of a German population survey. J Psychosom Res.

[28] R Core Team: A Language and Environment for Statistical Computing. Vienna, Austria: R Foundation for Statistical Computing. 2023. https://www.R-project.org [Access July 6 2023].

[29] Canavan C, West J, Card T (2014). The epidemiology of irritable bowel syndrome. Clin Epidemiol.

[30] Henningsen P, Gündel H, Kop WJ, Löwe B, Martin A, Rief W (2018). Persistent physical symptoms as perceptual dysregulation: a neuropsychobehavioral model and its clinical implications. Psychosom Med.

[31] Franke A, Singer MV, Dumitrascu DL (2009). How general practitioners manage patients with irritable bowel syndrome. Data from a German urban area. Rom J Intern Med.

[32] Hungin APS, Molloy-Bland M, Claes R, Heidelbaugh J, Cayley WE, Muris J (2014). Systematic review: the perceptions, diagnosis and management of irritable bowel syndrome in primary care– a Rome Foundation Working Team Report. Aliment Pharmacol Ther.

[33] Thompson WG, Heaton KW, Smyth GT, Smyth C (2000). Irritable bowel syndrome in general practice: prevalence, characteristics, and referral. Gut.

[34] U.S. Department of Health and Human Services, Office of Disease Prevention and Health Promotion. National action plan to improve health literacy. Washington, DC.; 2010. Available: https://health.gov/sites/default/files/2019-09/Health_Literacy_Action_Plan.pdf [Accessed Dec 28 2023].

[35] Schaeffer D, Hurrelmann K, Bauer U, Kolpatzik K. National Action Plan Health Literacy - Promoting health literacy in Germany. Berlin: Kompart, 2020. Available: https://www.nap-gesundheitskompetenz.de/ [Accessed Dec 28 2023].

[36] Ringström G, Störsrud S, Lundqvist S, Westman B, Simrén M (2009). Development of an educational intervention for patients with irritable bowel syndrome (IBS)– a pilot study. BMC Gastroenterol.

[37] Sierzantowicz R, Lewko J, Jurkowska G (2020). The impact of an individual educational program on the quality of life and severity of symptoms of patients with irritable bowel syndrome. Int J Environ Res Public Health.

