# Supplementary material for: Public knowledge and beliefs about the irritable bowel syndrome - results from the SOMA.SOC study
Source: BMC Public Health. 2024 Jan 18;24:219. doi: 10.1186/s12889-024-17784-8 (PMC10797779; doi:10.1186/s12889-024-17784-8)
Supplement: Supplementary file 1 — Supplementary Material 1 [file 12889_2024_17784_MOESM1_ESM.docx]

**Appendix: Irritable bowel syndrome vignettes used in the study**

Ten years ago, 37-year-old Gülsen E. came to Germany from Turkey and she works as a cleaner.^1^ For many months, she has been suffering from frequently recurring abdominal cramps, flatulence and diarrhoea. These troubles put a great strain on Ms. E.'s everyday life and her quality of life is severely restricted as a result. Ms. E. tells her doctor that she has not lost any weight, has no fever or blood in her stool. Examinations up to now have not provided any indication of a threatening disease.

37-year-old Martin E. works as a lawyer.^1^ For many months, he has been suffering from frequently recurring abdominal cramps, flatulence and diarrhoea. These troubles put a great strain on Mr. E.'s everyday life and his quality of life is severely restricted as a result. Mr. E. tells his doctor that he has not lost any weight, has no fever or blood in his stool. Examinations up to now have not provided any indication of a threatening disease.

^1^ Migration history (yes/no), sex (male/female), and occupational status (lawyer/cleaner) were systematically varied.
